# Cross-species global and subset gene expression profiling identifies genes involved in prostate cancer response to selenium

**DOI:** 10.1186/1471-2164-5-58

**Published:** 2004-08-20

**Authors:** Michael Schlicht, Brian Matysiak, Tracy Brodzeller, Xinyu Wen, Hang Liu, Guohui Zhou, Rajiv Dhir, Martin J Hessner, Peter Tonellato, Mark Suckow, Morris Pollard, Milton W Datta

**Affiliations:** 1 Department of Pathology, Medical College of Wisconsin, 8701 Watertown Plank Road, Milwaukee, WI, 53226, USA; 2 Department of Pathology, Winship Cancer Institute, Emory University School of Medicine, 1365-B Clifton Road NE, Atlanta, GA, 30322, USA; 3 Bioinformatics Program and Human and Molecular Genetics Center, Medical College of Wisconsin, 8701 Watertown Plank Road, Milwaukee, WI, 53226, USA; 4 Department of Pathology, University of Pittsburgh Medical Center, 200 Lothrop Street, Pittsburgh, PA, 15242, USA; 5 Department of Pediatrics and Human and Molecular Genetics Center, Medical College of Wisconsin, 8701 Watertown Plank Road, Milwaukee, WI, 53226, USA; 6 Department of Physiology, Medical College of Wisconsin, 8701 Watertown Plank Road, Milwaukee, WI, 53226, USA; 7 Walther Cancer Center, Lobund Laboratories, 400 Freiman Life Science Center, Notre Dame University, Notre Dame, IN, 46556, USA

## Abstract

**Background:**

Gene expression technologies have the ability to generate vast amounts of data, yet there often resides only limited resources for subsequent validation studies. This necessitates the ability to perform sorting and prioritization of the output data. Previously described methodologies have used functional pathways or transcriptional regulatory grouping to sort genes for further study. In this paper we demonstrate a comparative genomics based method to leverage data from animal models to prioritize genes for validation. This approach allows one to develop a disease-based focus for the prioritization of gene data, a process that is essential for systems that lack significant functional pathway data yet have defined animal models. This method is made possible through the use of highly controlled spotted cDNA slide production and the use of comparative bioinformatics databases without the use of cross-species slide hybridizations.

**Results:**

Using gene expression profiling we have demonstrated a similar whole transcriptome gene expression patterns in prostate cancer cells from human and rat prostate cancer cell lines both at baseline expression levels and after treatment with physiologic concentrations of the proposed chemopreventive agent Selenium. Using both the human PC3 and rat PAII prostate cancer cell lines have gone on to identify a subset of one hundred and fifty-four genes that demonstrate a similar level of differential expression to Selenium treatment in both species. Further analysis and data mining for two genes, the Insulin like Growth Factor Binding protein 3, and Retinoic X Receptor alpha, demonstrates an association with prostate cancer, functional pathway links, and protein-protein interactions that make these genes prime candidates for explaining the mechanism of Selenium's chemopreventive effect in prostate cancer. These genes are subsequently validated by western blots showing Selenium based induction and using tissue microarrays to demonstrate a significant association between downregulated protein expression and tumorigenesis, a process that is the reverse of what is seen in the presence of Selenium.

**Conclusions:**

Thus the outlined process demonstrates similar baseline and selenium induced gene expression profiles between rat and human prostate cancers, and provides a method for identifying testable functional pathways for the action of Selenium's chemopreventive properties in prostate cancer.

## Background

Gene expression profiling, along with other methods to evaluate the global changes in genomes, provides the opportunity to understand whole scale changes present in human biology. Yet the sheer mass of data presented by these techniques often makes subsequent analysis difficult. Techniques such as gene expression profiling may result in the identification of hundreds if not thousands of differentially expressed genes that may be associated with the biological process, but may also represent noise related to the biological and technical variation. In an economic environment where limited resources are available for the follow-up and validation of potential target genes methods must be provided for the prioritization and sorting of data. Previous methods have relied heavily on the mapping of metabolic pathways or transcription factor binding sites [1-5]. These processes rely on the premise that the metabolic pathways associated with a given disease are well delineated, or that groups of proteins with very similar structural or functional design are involved in the disease process. In situations where these assumptions may not be true, alternative methods for the sorting of the data are needed. Here we demonstrate an alternative approach using comparative genomics and animal models of human prostate cancer to sort and identify genes involved in the response of prostate cancer cells to the proposed chemopreventive agent Selenium [6,7]. This process takes advantage of the continued sequencing of multiple animal genomes and the ability to produce gene expression profiles in multiple species. Through the use of these techniques one can leverage established animal models to identify genes associated with human disease processes, as is demonstrated here with the identification of Insulin-like growth factor-2 Binding protein 3 (IGFBP3) and retinoid-X-receptor alpha (RXRalpha).

## Results

### Generation of common genes and homologs

Sequence validated gene libraries for both the rat and human DNAs were obtained from Research Genetics (Huntsville, AL), and were supplemented with additional DNA samples obtained from the University of Iowa rat clone sequencing program [8]. The majority of the rat DNAs, and a subset of the human DNAs were resequenced by Dr. J. Quackenbush at TIGR through a joint Program in Genomic Applications consortium. The GeneBank accession numbers for the 19,200 individual human or rat clones present in the recent slide printings were used to query the NCBI Unigene database to return the associated Unigene IDs. Unigene IDs were returned for virtually all identified clones, and were placed in an Oracle database where they were compared with the downloaded NCBI Homologene dataset (build 106) of rat, mouse, and human homologues. Of the 19,200 clones, 5740 genes were identified with homologues present on both the rat and human slides. This homologue set was used for the subsequent comparisons across species.

### Similar global and prostate gene expression profiles between rat and human prostate cancer cell lines

**Figure 1:**
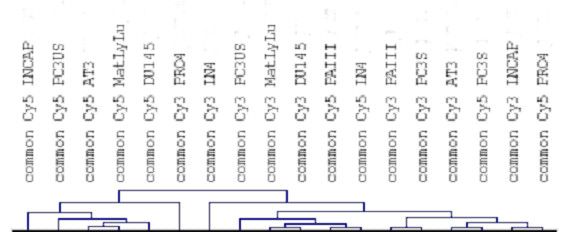
Gene expression profiles for human and rat prostate cancer cells. Clustering of the expressed genes in the human (LNCAP, DU145, PRO4, LN4, and PC3 derivatives) and rat (AT3, MatLyLu, and PAIII) prostate cancer cell lines based on the common homologs as defined within to NCBI Homologene database. Raw data files are available for review from the corresponding author.

**Figure 2:**
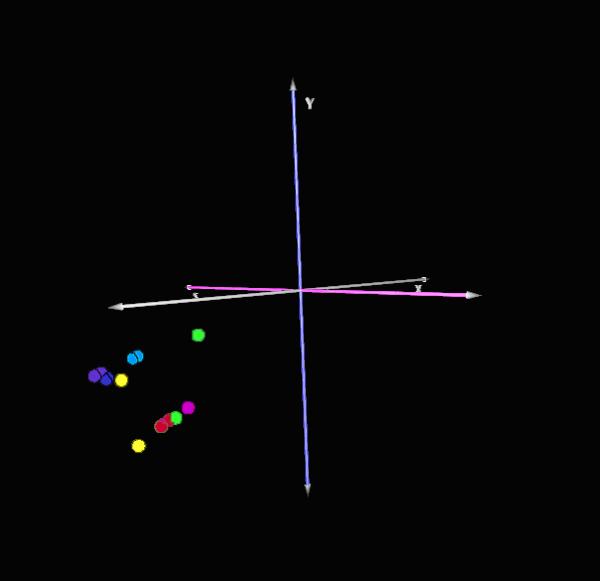
Principal Components Analysis of Rat and Human Prostate Cancer Cell Lines. There is a clustering of the human (Pro4-purple, LN4-dark-blue, PC3S-light blue, PC3US-yellow) and rat (MatLyLu-red, AT3-magenta, PAIII-green) prostate cancer cell lines in the same quadrant. The degree of separation within the quadrant was not significant by T-testing. Each sample is presented in duplicate based on independent Cy3 and Cy5 vector profiles.

We have sought to compare the rat and human prostate cancer transcriptomes in an effort to judge the degree of similarity between the two cell types. Because the use of differentially expressed genes would bias the comparison by eliminating the majority of genes that do not show any difference, we used the absolute level of expression for each gene and compared the rat and human genes for significant differences in absolute expression levels. In order to derive the absolute level of expression for individual genes in human or rat prostate cancer cells we used expression values derived from the associated self-self hybridizations performed for each cell line. The experiments were facilitated by the use of slides that have been quality controlled for the quantity of spotted target DNA through the use of a FITC label third dye [9]. These slides were subsequently imaged for FITC fluorescence and sorted based on the similar amounts of target DNA present on each slide [10]. Using the third dye quality control correlation coefficients of greater than 0.80 are routinely achieved between slide replicates [9]. In this manner comparisons of bound hybridized probe can be made across slides with a degree of confidence. RNA samples from cells were harvested, labeled, and homotypically hybridized to establish the baseline level of consistency within the hybridizations. Performing slice analysis on the normalized homotypic gene expression data across all the self-self hybridization slides within a species and retaining genes that demonstrated consistent expression patterns within two standard deviations of the mean expression value was performed to remove a degree of error from the technical replicates. Using the third dye as a baseline for comparison, these common expressed genes were then broken down into their component Cy3 or Cy5 expression vectors and used to build the transcriptomes for each gene using their absolute expression values. These transcriptomes were then used to compare expression values between the rat and human cell lines. These genes were annotated and gene homologues identified from the NCBI Homologene[11] dataset of rat-human homologues. Thus from a dataset of 5740 homologues, 2883 genes were found that were present within this experimental dataset and expressed in both the rat and human prostate cancer cell lines, and thus could be used for comparative genomics. These samples were processed using the Multiexperiment Viewer mircoarray statistical analysis and visualization program developed by TIGR [12]. Files were loaded and visualized for comparison across the 2883 common expressed genes in a self-organizing tree algorhythm [13] (figure 1) and analyzed for similarities in global expression patterns. The hierarchical clustering in self-organizing trees failed to demonstrate a pattern of clustering between species. T-test analysis [12,14] between the human and rat cell lines identified 58 genes (2%) which demonstrated significantly different expression patterns between species (p < 0.01 with Bonferroni correction). Thus in these comparisons, 2826 genes, or 98% of the genes examined, failed to demonstrate a statistically significant difference in expression between the human and rat prostate cancer cell lines. Using principle components analysis (figure 2, [12]) these studies can be visualized, and demonstrate while there is some clustering of the rat and human prostate cancer cell lines, the differences are not significant. Thus when comparing gene expression patterns in rat and human cell lines one will detect significant species-specific differences in expression in 1 out of every 50 genes, with the majority of the genes demonstrating similar expression patterns.

The presence of a large quantity of genes whose expression may be related to general cellular functions, as opposed to prostate specific metabolism, could infuse a significant amount of homogeneity to the data. In the presence of such homogeneity it may be impossible to identify the true differences that are related to prostate cellular function, and thus the perceived similarities may be artifactual. To address this issue we sought to repeat the analysis using only prostate related genes. To generate a list of such genes we used cDNAs in eight normal human prostate cDNA libraries present in the NCI Cancer Genome Anatomy Project [15]. Generation of a list of common genes proved impossible, as the combination of more than four of the cDNA lists resulted in the number of common genes being reduced to zero. A similar result was obtained when one attempted to generate a list of commonly expressed genes across multiple different cancer cDNA libraries. As an alternative approach we developed a list of 12,008 expressed genes were identified based on their presence in at least one of the eight normal human prostate cDNA libraries. The human Unigene IDs for each of the expressed genes were then used to identify the associated rat homologues from Homologene [11] and yielded 2,269 homologous rat genes (18.9%), of which 1,319 (58.1%) had associated prostate cancer gene expression data. These 1,319 prostate expressed genes were then used to repeat the comparative genomics. Similar visual and clustering results were identified for the prostate transcriptomes. T-test analysis [12,14] between the human and rat cell lines identified 30 prostate expressed genes (2%) which demonstrated significant differential expression between species (p < 0.01 with Bonferroni correction, while 1,289 genes (98%) failed to demonstrate a significant difference in expression across species. Thus even when only prostate expressed genes are considered, similar results were obtained. Between the rat and human prostate cancer cell lines the patterns of expression are similar for 49 of 50 genes examined.

### Comparison of global and prostate specific differential gene expression profiles between rat and human prostate cancer cell lines treated with selenium

While global gene expression profiles appear to be similar between rat and human prostate cancer cell lines one wonders whether the response to specific physiologic stimuli may elicit similar transcriptional changes. If so, one may be able to infer a degree of homology in their biological response to the stimuli. This has already been observed on a physiological level for the rat models of prostate cancer. For example, rat and human prostate cancers respond very similarly to chemotheraputic and environmental agents including hormonal agents (both respond), cyclophosphamide (neither respond), high fat diets (increased incidence), and soy isoflavones (decreased incidence) [16-22]. In an effort to evaluate these similar biological responses we have compared the transcriptomes between rat and human prostate cancer cell lines treated with the proposed prostate cancer chemopreventive agent Selenium. Samples from the human PC3 and rat PA-III cell lines were treated with Selenium and examined for differential gene expression profiling. These two cell lines were chosen based on their similar biologic characteristics, as both cell lines were derived from androgen independent metastatic tumors, and thus represent tumors with similar biologic potential [23,24]. The cells were treated with twenty-five micromolar Selenium for either 6 hours or 5 days, to identify both immediate changes in gene transcription or changes related to the long term exposure to Selenium. Due to our interest in prostate cancer we have attempted to choose a form and concentration of Selenium that would be reflected in the ongoing prevention trials such as the SELECT prostate cancer prevention trial [25,26]. In this trial patients receive Selenium in the form of Selenized baker's yeast. Previous HPLC and electrospray mass spectroscopy studies have demonstrated that 85% of the Selenium in yeast is present as selenomethionine [27]. Selenomethionine has previously been used in *in-vitro *studies of prostate cancer cells[28,29]. These studies demonstrated an inhibition of prostate cancer cell proliferation over a broad range of concentrations, while an IC50 and/or decreased expression was seen at concentrations above 70 micromolar selenomethionine. To avoid the general effects of cell inhibition or cell death while focusing on the effect of Selenium we chose a lower concentration of 25 micromolar selenomethionine. These changes, while not resulting in increased cell death, did cause decreased cell division and increased doubling time in both species (data not shown). Common rat and human homologous genes demonstrating differential expression by greater than two standard deviations were identified and included 1123 genes after 6 hours and 1053 genes after 5 days of exposure to Selenium. When the expression patterns of these genes were compared across species by T-test and principle component analysis as outlined above 713 genes (25%) were found to have statistically significant differences in expression between species (p < 0.01 with Bonferroni correction). Thus when comparing rat and human samples, while the majority of the gene expression changes are similar, in at least one in four genes (p = 0.75) one can detect significant species specific differences in expression alteration when cells are treated with Selenium. Yet similar physiologic changes (decreased cellular proliferation, increased cell death) were observed in both species. These changes represent the desired physiologic changes one would expect for the chemopreventive effects of Selenium, and could be dissected by examining the common transcriptional changes seen in both species with respect to Selenium.

### Combined differential expression patterns for selenium responsive genes identify common gene pathways

Because some of the differences in the rat and human prostate cancer cell line transcriptomes may be related to confounding variables such as culture methods, cell passage number, or time in culture, an effort was made to focus on genes that are common, and as such may define the similar Selenium based cell proliferative changes. The subsets of 1123 and 1053 differentially expressed genes (6 hours and 5 days respectively) were analyzed for genes that demonstrate similar changes in expression with respect to Selenium across species. Of these differentially expressed genes, 291 and 309 demonstrated up-regulation in rat and human cells at 6 hours and 5 days respectively. Likewise, 261 (6 hours) and 216 (5 days) demonstrated down-regulation in the presence of Selenium. When these subsets were further analyzed to identify genes with similar levels of up or down-regulation (defined as ratio differences within 0.2 units of each other) 81 genes were identified at 6 hours and 73 at 5 days (table 1-see additional file 1). These genes included 40 ESTs or genes with limited associated data, and 90 defined genes with associated gene data. Twenty-four of the genes were common to Selenium treatment at both 6 hours and 5 days. Additional information related to these genes was obtained using the GeneInfo data mining tool. This tool was developed by the authors (MWD, XW, HL, GZ) to allow for the rapid identification of supplemental data from the biomedical literature related to genes of interest. In brief, the tool allows one to cut and paste a list of genes based on either Unigene or Genebank IDs and search PubMed for associated references based on annotations of the associated gene names. Additional search terms can be stipulated by the user based on their knowledge of the biological process or in response to results received from the previous search. Results are returned in a table that lists the number of references that met the search criteria and provides a hyperlink to the associated references for either downloading or viewing. In this way the user is allowed to direct queries in an open manner based on their own experience or unpublished data. In this manner searches were conducted using the list of genes and the search terms "prostate cancer", "Selenium", and "apoptosis" (table 1-see additional file 1).

### IGFBP3 and RXR-alpha are expressed in the prostate, induced by selenium, and downregulated in prostate cancer

**Figure 3:**
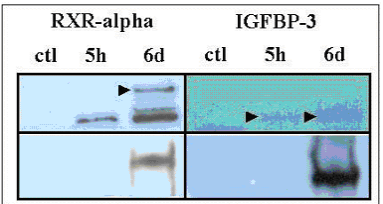
Expression of IGFBP3 and RXR-alpha with respect to Selenium. Western blotting reveals an induction of RXR-alpha or IGFBP-3 protein after Selenium treatment of human PC3 prostate cancer cells (arrows, upper row). Western blotting of immunoprecipitations from rat PAIII cells (bottom row) reveal RXR-alpha in immunoprecipitated IGFBP3 extracts (right panel) and IGFBP-3 in immunoprecipitated RXR-alpha extracts confirming and extending the reported interactions between the human proteins[40].

**Figure 4:**
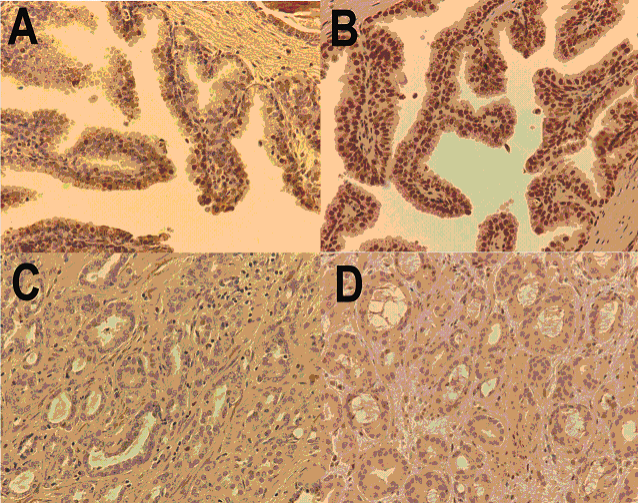
Expression of IGFBP3 and RXRalpha in human prostate tissues. Immunohistochemical staining for IGFBP3 is present as brown staining in normal prostate (A) and prostate cancer (C). Similarly RXRalpha expression is present in normal prostate (B) and lost in prostate cancer (D). All images recorded at 100× magnification.

Of the 154 genes identified with similar cross-species differential expression changes with respect to Selenium, two genes were identified that had unique features based on their associated references and interrelated functions. These genes, IGFBP3 and RXR-alpha were both up-regulated with respect to Selenium and could be used to suggest a model for Selenium action in prostate cancer. PXR-alpha is upregulated in both rat and human prostate cancer cells at 5 days in response to Selenium. Likewise, IGFBP3 is upregulated after six hours of Selenium treatment in both species. These two genes both contained Medline references with respect to prostate cancer, but had not yet been implicated in Selenium action. Western blotting performed on the human prostate cancer cell line PC3 with respect to Selenium validated the bioinformatically identified expression data (figure 3). To confirm the role of these two proteins in the prostate immunohistochemical studies on prostate cancer tissue microarrays were performed to identify IGFBP3 and RXR-alpha in both normal, nodular hyperplasia (benign prostatic hypertrophy), high grade prostatic intraepithelial neoplasia (HGPIN), invasive carcinoma, and metastatic prostatic carcinoma (table 2). These studies demonstrate that both IGFBP3 and RXR-alpha are expressed in the normal human prostatic epithelium (figure 4, table 2). IGFBP3 is also expressed in the prostatic basal cells. Patterns of expression were predominantly nuclear, a finding that has been described for both proteins [30]. In addition, staining for IGFBP3 was also noted in the prostatic stroma, consistent with IGFBP3's associated function as a secreted protein. Decreased levels of IGFBP3 was noted in prostatic cancers when compared to normal prostate epithelium (p = 0.0044). Along with this decreased expression there was a distinct shift in the protein localization nuclear to cytoplasmic was observed (p < 0.00001), and in cases where expression was still present, there were decreased numbers and intensity of cell staining. IGFBP3 expression was similar in HGPIN, invasive carcinoma, and metastatic carcinoma. The level and pattern of IGFBP3 expression in nodular hyperplasia was similar to that seen in normal prostate tissues, and significantly different from the expression seen in cancer samples (p = 0.0036 and p < 0.00001 respectively). RXR-alpha expression was also significantly downregulated in prostate cancer when compared to normal prostate epithelium or nodular hyperplasia (p < 0.0001), and was similar to that seen in HGPIN and metastatic carcinoma. RXR-alpha expression was consistently nuclear in the samples studied, and while the intensity of staining was similar, in the remaining positive cancer cases there were decreased numbers of cells staining (8.6 +/- 12.6% in malignant epithelium vs 20.0 +/- 25.5% in normal epithelium).

**Table 2:** Expression of IGFBP3 and RXRalpha in Prostatic Epithelium

	Normal Prostate	Nodular Hyperplasia	HGPIN	Prostate Cancer	Metastatic Cancer
IGFBP3					
Positive cases	105	62	49	202	25
Negative cases	5	1	9	36	8
Statistics (comparison)		p = 0.0036 (cancer)	N.S. (cancer)	p = 0.0044 (normal)	N.S. (cancer)
IGFBP3					
Intensity (avg+/-std)	2.47 +/- 0.70	2.49 +/- 0.65	2.57 +/- 0.82	2.74 +/- 0.56	2.79 +/- 0.49
Percentage cells (avg+/- std)	8.3 +/- 13.5	7.5 +/- 12.5	8.8 +/- 15.2	4.4 +/- 6.6	8.5 +/- 12.6
Nuclear cases	92	59	40	94	8
Cytoplasmic cases	22	6	18	152	11
Statistics (comparison)		p < 0.00001 (cancer)	p = 0.065 (cancer)	p < 0.00001 (normal)	N.S. (cancer)
RXRalpha					
Positive cases	92	58	35	112	16
Negative cases	10	3	31	125	19
Statistics (comparison)		p < 0.00001 (cancer)	N.S. (cancer)	p < 0.00001 (normal)	N.S. (cancer)
RXRalpha					
Intensity (avg+/-std)	2.73 +/- 0.51	2.78 +/- 0.50	2.83 +/- 0.38	2.76 +/- 0.49	3 +/- 0
Percentage cells (avg+/- std)	20.0 +/- 25.5	23.2 +/- 25.7	8.4 +/- 12.5	8.6 +/- 12.6	4.2 +/- 4.6
Nuclear cases	92	58	35	107	16
Cytoplasmic cases	2	0	6	9	0
Statistics (comparison)		N.S. (cancer)	N.S. (cancer)	N.S. (normal)	N.S. (cancer)

## Discussion

### Leveraging cross-species bioinformatics in the prioritization of gene data

Through the use of cross-species comparisons of the number of differentially expressed genes to be examined after 6 hours and 5 days of Selenium treatment was dropped from 9453 and 7768 to 1123 and 1053 respectively, an 87–89 percent reduction of the sample size. Even with the use of multiple timepoints, the number of differentially expressed genes was only reduced in a single species study to 5934, less than half. By using comparative genomics the final dataset was reduced to 154 genes, providing a greater than 100 fold enrichment of the data. Thus by leveraging the additional biological species the ability to reduce the final analysis pool was substantial. This process only works if the species used have biological relevance to the disease in question. The choice of rat prostate cancer cell lines was made based on their use as an animal model for the study of prostate cancer [31]. The animal systems have been extensively used in the study of hormonal carcinogenesis, and in particular have been of value as a model of environmental and dietary effects on prostate cancer [18-20]. Previous studies have identified similar effects of rat animal models and prostate cancer cell lines to soy based diets [17-19], high fat diets [20-22], hormonal chemotherapeutics (Pollard, personal communication) and standard chemotherapy [32,33]. While comparative gene expression profiling has been performed, this has usually been through cross-species hybridizations to leverage RNA studies in species where sufficient expressed transcripts in a given species have not been identified for the production of species-specific gene expression slides, in particular for microbial genomes [34-37]. Thus the approach taken here leverages the production of species-specific gene expression profiles along with the increasing amount of gene homolog data generated by the sequencing of additional animal genomes. It is expected that with future genome efforts additional cross-species studies will be possible that leverage the knowledge of additional animal models in the study of disease.

### Similarities in prostate cancer transcriptomes across species

For both overall and prostate expressed genes, we have failed to identify a significant difference in the transcriptomes between rat and human prostate cancer cell lines. This general similarity in transcriptomes may be due to the inherent biological similarities of the cell lines and/or their underlying biological origin. While the studies sought to utilize prostate cancer cell lines with similar biological potentials (established cell lines all derived from metastases) the degree of diversity present within the samples may account for some of the residual differences still identified. In addition, the extended period of time that these cell lines have been used has allowed for the continued in-vitro evolution of the cells, and could possibly extend those genomic differences. Yet the common clustering of the rat and human cell lines together suggests there are still significant similarities in their biological potential. This is also demonstrated by the similar biological potential of the cell lines when treated with a given stimulus, in this example Selenium. This parallels the similar physiological properties observed in the rat models of human prostate cancer. Based on these features we demonstrate that it is possible to identify functionally significant genes related to Selenium response by using comparative genomics. These findings also support the use of animal models in the study of human prostate cancer by suggesting that there is enough inherent genomic similarity that valuable insights may be gained from animal systems.

### Comparative genomics identifies functionally significant genes with respect to selenium chemoprevention

A true test of the profiling method is the identification of genes that have a functional significance to the experimental system. In this case we have identified a series of genes, which when examined with additional data mining techniques, identifies genes with associated roles related to apoptosis (IGFBP3, RXRalpha, dynamin-2), antioxidant protection (selenoprotein N, peroxiredoxin I, zinc metalloprotease, glutathione S transferase), cell cycle (CDC26-anaphase promoting complex, kinetochore associated protein), and protein balance (proteasome subunit beta-4, ubiquitin conjugating enzyme). In addition, the ability to sort the identified genes by their associated biomedical literature allowed the focus to shift to IGFBP3 and RXRalpha. Retinoids, through the retinoid X receptor, have been shown to induce the expression of IGFBP3 [38]. In concert these two proteins act to induce apoptosis in cancer cell lines [39]. In particular, recent data has shown that these proteins work in synergy to enhance apoptosis in prostate cancer, and that there is a physical interaction between these two proteins in prostate cancer cells[40]. Further validation and confirmatory data is presented here that demonstrates the selenium induced expression and interaction between both RXRalpha and IGFBP3 in prostate cancer cells, along with their expression in normal prostate epithelium and subsequent down-regulation in malignant prostatic epithelium. This allows one to pose a model by which the restoration of IGFBP3 and RXRalpha levels by Selenium treatment may lead to the disruption of prostate tumorigenesis. This model is testable, and if validated, would present not only a mechanism by which Selenium may exert its effect, but provide a biomarker for assaying the effect of Selenium supplementation in the ongoing prostate cancer prevention clinical trials.

## Conclusions

Using gene profiling on highly controlled spotted cDNA arrays we have demonstrated that similar baseline and selenium induced gene expression profiles can be identified between rat and human prostate cancer cells. This has allowed us to filter our gene expression data to identify genes whose transcriptional response to Selenium is similar across species, and by so doing focus our discovery process on specific common physiologic pathways. Two such proteins, RXR-alpha and IGFBP-3, which may be located in a common pathway, have been identified as dysregulated in human prostate cancers. This provides further support that the cross-species methods employed here can identify genes with roles in human prostate cancer.

## Methods

### Cell culture and selenium treatment

Cell lines were received from ATCC, Rockford, MD, (LNCap, DU-145, MatLyLu, AT3), from Drs. Paul Lindholm and Andre Kadjacsy-Balla (LN4, Pro4, PC3, PC3-NI(PC3US), PC3-I(PC3-S)), or Dr. Morris Pollard and Mark Suckow (PA-III). These cells were cultured in RPMI (DU-145) or DME medium supplemented with 10% fetal calf serum, 10 mM glutamine, and 10 mM sodium pyruvate, and passaged 1:8 or 1:10 when the cells reached 70–80% confluence with trypsin-EDTA. For the Selenium studies PC3 or PAIII cells from a single cell stock were seeded at 1 × 10EE4 cells per ml and grown to 50% confluence at which time the culture medium was changed to either standard growth medium (above) or medium supplemented with twenty-five micromolar Selenium (Seleno-DL-methionine, Sigma cat# S3875, St. Louis MO). The cells were then cultured for an additional 6 hours or 5 days. Cells that reached 80% confluence prior to the five day timepoint were split using trypsin-EDTA and replated in either control or selenium-containing medium for the duration of the experiment. Cells were monitored for viability and cell growth with parallel growth curves conducted in triplicate, this data demonstrated the previously described [41,42] decrease in cellular proliferation (data not shown) observed in the presence of Selenium.

### RNA isolation and quantitation

RNA was isolated from cells using Trizol (Invitrogen cat # 15596018, Carlsbad, CA) and subsequently examined for quality using agarose gel electrophoresis and Gelstar nucleic acid stain against known RNA standards and failed to demonstrate significant degradation based on the presence of high molecular weight RNA species, and intact 28s and 18s ribosomal RNA bands.

### DNA library preparation and amplification

Sequence-verified rat and human libraries (Research Genetics, Huntsville, AL, and University of Iowa cDNA clone set, IA), consisting of 41,472 human clones and 36,000 rat clones were used as a source of probe DNA. A subset of 200 randomly selected clones were chosen from these libraries, resequenced locally, and demonstrated clone accuracy of 92%. We have opted to reformat libraries from 96 to 384-format for culture growth/archiving, PCR, purification, and printing. This has reduced the number of plates of our 41,472 human clone library from 432 to a more manageable 108, and the rat clone library from 375 to 94. The library was reformatted and subsequently manipulated using slot pin replicator tools (VP Scientific, San Diego, CA). Cultures were grown in 150 ul Terrific Broth (Sigma, St. Louis, MO) supplemented with 100 mg/ml ampicillin in 384 deep-well plates (Matrix Technologies, Hudson, NH) sealed with air pore tape sheets (Qiagen, Valencia, CA) and incubated with shaking for 14–16 hr. Clone inserts were amplified in duplicate in 384-well format from 0.5 μl bacterial culture diluted 1:8 in sterile distilled water or from 0.5 μl purified plasmid (controls only) using 0.26 μM of each vector primer {SK865 5'-fluorescein-GTC CGT ATG TTG TGT GGA A-3' and SK536: 5'-fluorescein-GCG AAA GGG GGA TGT GCT G-3'} (Integrated DNA Technologies, Coralville, IA) in a 20 μl reaction consisting of 10 mM Tris-HCl pH8.3, 3.0 mM MgCl_2_, 50 mM KCl, 0.2 mM each dNTP (Amersham, Piscataway, NJ), 1 M betaine, and 0.50 U *Taq *polymerase (Roche, Indianapolis IN). Reactions were amplified with a touchdown thermal profile consisting of 94°C for 5 min; 20 cycles of 94°C for 1 min, 60°C for 1 min (minus 0.5° per cycle), 72°C for 1 min; and 15 cycles of 94°C for 5 min; 20 cycles 94°C for 1 min, 55°C for 1 min, 72°C for 1 min; terminated with a 7 min hold at 72°. PCR reactions analyzed for single products by 1% agarose gel electrophoresis analysis. Products from replicate plates were pooled and then purified by size exclusion filtration using the Multiscreen 384 PCR filter plates (Millipore, Bedford, MA) to remove unincorporated primer and PCR reaction components. Forty wells of each 384-well probe plate were quantified by the PicoGreen assay (Molecular Probes, Eugene, OR) according to the manufacturers instructions. After quantification, all plates were dried down, and reconstituted at 150 ng/μl in 3% DMSO/1.5 M betaine.

### Array slide fabrication

A single printing array containing 19,200 elements (human) or 2 arrays of 9,600 (rat), were printed on poly-L-lysine coated slides prepared in-house (1–2 arrays/slide) as previously described [9]. Printing was conducted with a GeneMachines Omni Grid printer (San Carlos, CA) with 16 or 32 Telechem International SMP3 pins (Sunnyvale, CA) at 40% humidity and 22°C. To control pin contact force and duration, the instrument was set with the following Z motion parameters, velocity: 7 cm/sec, acceleration: 100 cm/sec^2^, deceleration: 100 cm/sec^2^. All slides were post-processed using the previously described nonaqueous protocol[9]. Slide coating was performed as described previously [43]. Image files on all arrays were collected after blocking (fluorescein), and again after hybridization (Cy3 and Cy5) with a ScanArray 5000 (GSI Lumonics, Billerica, MA).

### Experimental design and bioinformatics based data analysis

The experimental design utilized two biological replicates for each comparison with each replicate incorporating a Cy3/Cy5 dye flip. In addition, self-self hybridizations were performed for each sample to ensure experimental accuracy and evaluate expression bias. Comparisons were organized in a loop design for either human or rat prostate cancer cell lines, or were run as two-sample comparisons of baseline untreated control and Selenium treated cells. Array image TIFF files were analyzed with Gleams software (Nutec Sciences, Atlanta, GA). Additional TIFF file analysis, data normalization, clustering, and principle components analysis was performed using the Spotfinder, MIDAS and MultiExperiment Viewer Software from The Institute for Genomic Research (TIGR, Rockville, MD, [44], [12]) and used default values set in the MCW Practical Guide to TIGR Software Use (M. Datta, unpublished). In brief, image expression data was used as channel intensity minus background and intensity thresholds were set at a value of 300. Images were analyzed as dye flip pairs normalized using MIDAS with LocFit based LOWESS normalization and slice analysis set at two standard deviation cutoffs and a sample data population of 500 [45]. Samples were then averaged across two dye flip replicate pairs with removal of zero/dropped values using locally developed averaging software from the BEAR microarray suite (M. Datta, submitted). These final averaged values were subsequently annotated using the BEAR suite annotator and used for pattern identification and correlation with gene homologs. Homologous genes were identified from the NCBI homologene database ftp files  and parsed using local scripts and databases present in the Bioinformatics Program,[46]. Additional data mining to identify references in the biomedical literature associated with specific genes and user chosen search terms was performed using the locally developed GeneInfo data tool (M. Datta, submitted). Raw data files, along with analyzed data subsets are available for use and study and can be obtained via a secure ftp site after contacting the corresponding author mdatta@mcw.edu.

### Protein purification, western blotting, and immunoprecipitation

Protein extracts were prepared and immunoprecipitations and/or western blots made from five day twenty-five micromolar Selenium treated or control PC3 or PAIII prostate cancer cell lines as described previously[47]. In brief, ten micrograms of total protein were run on pre-cast 12% reducing SDS PAGE gels (Bio-Rad Labs, Hurcules, CA) and transferred to PVDF membranes. After blocking with caseine blocking buffer (Bio-Rad Labs, Hurcules, CA) the PVDF membranes were incubated with either anti-RXR-alpha or anti-IGFBP-3 antibodies (Santa Cruz Biotechnology, Santa Cruz, CA) at 200 μg/ml dilutions, washed, and incubated with anti-rabbit secondary antibody (2 μg/ml) and developed with ECL Chemiluminescence (cat. RPN2108, Amersham Biosciences, Piscataway, New Jersey). Immunoprecipitations were carried out using 200 microgram samples of total cellular protein, which after preclearing with protein A agarose beads was sequentially incubated with either anti-RXR-alpha (1 μg/ml) or anti-IGFBP-3 (1 μg/ml) antibodies, washed, incubated with anti-rabbit protein A agarose beads, washed, and the protein pellet western blotted with the complimentary antibody (anti-IGFBP-3 or anti-RXR-alpha, respectively), and developed with ECL Chemiluminescence.

### Tissue microarray production, immunohistochemistry, and analysis

After expedited institutional review board approval normal prostate tissues and prostate cancer samples were obtained from de-identified discarded patient specimens. The formalin-fixed paraffin embedded specimens were prepared as 5 micron sections. Tissue microarrays were prepared from donor tissue blocks as 0.6 mm cores in 12 (4 × 4) or (5 × 5) grids with between 192 to 300 samples and used in the preparation of 5 micron sections. Immunohistochemistry was performed using primary rabbit polyclonal antibodies to the insulin-like growth factor binding protein 3 (IGFBP3, 1:300, Santa Cruz Biotechnology, Santa Cruz, CA), or retinoic-X-receptor alpha (RXR-alpha, 1:800, Santa Cruz Biotechnology, Santa Cruz, CA) using methods previously described [48,49]. In brief, endogenous peroxidase from deparaffinized sections were blocked with Methanol/Acetic acid, and after treatment with blocking serum (ABC kit, Pierce Biotechnology, Rockford, IL) samples were incubated for 30 minutes with either anti-IGFBP3 (1:300) or anti-RXRalpha (1:600). Sections were subsequently washed, and incubated with anti-rabbitt secondary antibody conjugated to horseradish peroxidase and counterstained with Mayers hematoxalin. Antigen retrieval (90 C waterbath for 10 minutes) was used for RXRalpha. Positive controls for each antibody included nuclear staining in Sertoli cells [50] and lymphocytes[51]. Positive staining was recorded and scored on a 0–2 scale (0 = no staining, 1 = staining that does not obscure the hematoxalyn counterstain, 2 = staining that obscures the hematoxalyn counterstain). Evidence of positive staining was recorded as presence of staining (yes/no) or percent of epithelial or basal cells staining (number of cells staining over total number of cells). Patterns of staining (nuclear, cytoplasmic, membranous, diffuse extracellular) were also recorded. All samples were analyzed and recorded by two separate personnel, including a trained urologic pathologist (MWD, BM). Statistical analysis was performed using Chi-squared probability analysis.

## Abbreviations

None declared.

## Authors contributions

M.W.D. was responsible for the conception and implementation of this project in association with P.J.T., M.S., and M.P. H.L., X.W., and G.Z. were actively involved in the programming, database construction, and testing of the software. M.S. and M.H. were responsible for spotted cDNA construction, hybridization, and experimental analysis along with M.W.D. Cell culture, western blots, immunoprecipitations, and selenium treatments were performed by M.S. with assistance by B.M. Tissue microarray staining and analysis was performed by M.W.D., R.D., T.B., and B.M. All the authors reviewed and accepted the final version of the paper.

## Supplementary Material

Additional File 1 Table 1, Word document, Table of the genes identified in the selenium gene expression studies. Click here for file
