# Supplementary material for: Cross-species global and subset gene expression profiling identifies genes involved in prostate cancer response to selenium
Source: BMC Genomics. 2004 Aug 20;5:58. doi: 10.1186/1471-2164-5-58 (PMC516028; doi:10.1186/1471-2164-5-58)
Supplement: Additional File 1 — Table 1, Word document, Table of the genes identified in the selenium gene expression studies. [file 1471-2164-5-58-S1.doc]

Table 1: GeneInfo Data Mining for Prostate Cancer

| Unigene | Name | State (both rat and human) | Prostate cancer | Selenium | Apoptosis |
| --- | --- | --- | --- | --- | --- |
| Hs.82587 | phospholipase D1, phophatidylcholine-specific | down 6 hours | 0 | 0 | 0 |
| Hs.100960 | KIAA0608 protein | down 5 days | 0 | 0 | 0 |
| Hs.107139 | EST | up 6 hours | X | X | X |
| Hs.108636 | chromosome 1 open reading frame 9 | down 5 days | 0 | 0 | 0 |
| Hs.109058 | ribosomal protein S6 kinase, 90kDa, polypeptide 5 | up 6 hours | 1 | 0 | 9 |
| Hs.109494 | secreted protein of unknown function | down 5 days | 0 | 0 | 0 |
| Hs.109727 | mitogen-activated protein kinase kinase kinase 7 interacting protein 2 | up 6 hours | 0 | 0 | 2 |
| Hs.110445 | CGI-97 protein | up 5 days | 0 | 0 | 0 |
| Hs.112028 | Misshapen/NIK-related kinase | down 5 days | 0 | 0 | 0 |
| Hs.112227 | EST | up 6 hours | X | X | X |
| Hs.115242 | developmentally regulated GTP binding protein 1 | up 5 days | 0 | 0 | 5 |
| Hs.115778 | kinetochore associated 1 | down 5 days | 0 | 0 | 0 |
| Hs.118354 | EST | up 5 days | X | X | X |
| Hs.11896 | hypothetical protein FLJ12089 | down 5 days | 0 | 0 | 0 |
| Hs.119018 | transcription factor NRF | down 6 hours | 0 | 0 | 4 |
| Hs.1211 | acid phosphatase 5, tartrate resistant | down 5 days | 11 | 0 | 32 |
| Hs.12144 | EST | down 6 hours | X | X | X |
| Hs.1216 | actinin, alpha 3 | up 6 hours | 0 | 1 | 13 |
| Hs.12451 | echinoderm microtubule associated protein like 1 | down 6 hours | 0 | 0 | 0 |
| Hs.12646 | hypothetical protein FLJ22693 | down 6 hours & 5 days | 0 | 0 | 0 |
| Hs.12705 | hypothetical 43.1 kd protein | down 6 hours & 5 days | 0 | 0 | 0 |
| Hs.13144 | EST | up 6 hours | X | X | X |
| Hs.14832 | decapping enzyme hDcp1b | down 6 hours | 0 | 0 | 0 |
| Hs.150926 | fucose-1-phosphate guanylyltransferase | up 6 hours | 0 | 0 | 0 |
| Hs.1513 | interferon (alpha, beta and omega) receptor 1 | up 6 hours | 0 | 0 | 0 |
| Hs.152925 | KIAA1268 protein | down 5 days | 0 | 0 | 0 |
| Hs.153636 | far upstream element (FUSE) binding protein 3 | down 6 hours & 5 days | 0 | 0 | 0 |
| Hs.15384 | AP1 gamma subunit binding protein 1 | down 5 days | 0 | 0 | 0 |
| Hs.15386 | EST | down 6 hours | X | X | X |
| Hs.154654 | cytochrome P450, subfamily I | up 6 hours | 1 | 0 | 1 |
| Hs.155040 | zinc finger protein 217 | up 5 days | 0 | 0 | 0 |
| Hs.155418 | GS3955 protein | up 5 days | 0 | 0 | 0 |
| Hs.155751 | ATP synthase, H+ transporting, | up 6 hours | 0 | 0 | 0 |
| Hs.16081 | chromodomain protein, Y chromosome-like | down 6 hours | 0 | 0 | 0 |
| Hs.167013 | dynamin 2 | down 6 hours & 5 days | 2 | 0 | 7 |
| Hs.168103 | prp28, U5 snRNP 100 kd protein | down 5 days | 0 | 0 | 0 |
| Hs.168212 | EST | down 6 hours | X | X | X |
| Hs.169965 | EST | up 6 hours | X | X | X |
| Hs.180909 | peroxiredoxin 1 | down 6 hours & 5 days | 1 | 8 | 19 |
| Hs.182215 | ADP-ribosylation factor-like 3 | down 5 days | 0 | 0 | 0 |
| Hs.182877 | KIAA0116 protein | up 6 hours | 0 | 0 | 0 |
| Hs.182877 | KIAA0116 protein | down 5 days | 0 | 0 | 0 |
| Hs.183171 | hypothetical protein FLJ22002 | down 5 days | 0 | 0 | 0 |
| Hs.184 | advanced glycosylation end product-specific receptor | up 5 days | 0 | 0 | 0 |
| Hs.184325 | ubiquitin-conjugating enzyme E2, J1 | up 5 days | 0 | 0 | 0 |
| Hs.18508 | glycine-N-acyltransferase | up 6 hours | 0 | 0 | 0 |
| Hs.18571 | protein kinase, interferon-inducible double stranded RNA dependent activator | down 5 days | 0 | 0 | 0 |
| Hs.19122 | eukaryotic translation initiation factor 4E-like 3 | down 6 hours & 5 days | 0 | 0 | 0 |
| Hs.191598 | EST | down 6 hours | X | X | X |
| Hs.19699 | Conserved gene telomeric to alpha globin cluster | down 6 hours & 5 days | 0 | 0 | 0 |
| Hs.19999 | DKFZP566K023 protein | down 6 hours | 0 | 0 | 0 |
| Hs.2006 | glutathione S-transferase M3 | down 6 hours | 0 | 0 | 0 |
| Hs.20084 | retinoid X receptor, alpha | up 5 days | 26 | 3 | 143 |
| Hs.2030 | thrombomodulin | down 5 days | 6 | 1 | 14 |
| Hs.21223 | calponin 1 | up 5 days | 3 | 0 | 6 |
| Hs.21263 | suppressor of potassium transport defect 3 | down 6 hours & 5 days | 0 | 0 | 0 |
| Hs.22129 | hypothetical protein DJ1042K10.2 | down 6 hours | 0 | 0 | 0 |
| Hs.23111 | phenylalanine-tRNA synthetase-like | down 6 hours | 0 | 0 | 0 |
| Hs.23450 | mitochondrial ribosomal protein S25 | up 6 hours | 0 | 0 | 0 |
| Hs.236218 | tripartite motif-containing 32 | down 6 hours | 0 | 0 | 0 |
| Hs.238272 | inositol 1,4,5-triphosphate receptor, type 2 | down 5 days | 0 | 0 | 0 |
| Hs.239506 | mab-21-like 1 | up 5 days | 0 | 0 | 0 |
| Hs.25732 | eukaryotic translation initiation factor 4 gamma, 3 | up 6 hours & 5 days | 7 | 0 | 3 |
| Hs.25846 | zinc metalloproteinase | up 5 days | 7 | 0 | 7 |
| Hs.26239 | hypothetical protein FLJ31810 | up 5 days | 0 | 0 | 0 |
| Hs.26395 | erythrocyte membrane protein band 4.1-like 1 | down 6 hours & 5 days | 0 | 0 | 0 |
| Hs.26981 | hypothetical protein MGC16131 | up 6 hours | 0 | 0 | 0 |
| Hs.2799 | cartilage linking protein 1 | up 6 hours & 5 days | 0 | 0 | 3 |
| Hs.29403 | hypothetical protein FLJ22060 | down 5 days | 0 | 0 | 0 |
| Hs.29640 | reversion-inducing-cysteine-rich protein with kazal motifs | up 6 hours | 0 | 0 | 0 |
| Hs.29692 | EST | up 6 hours | X | X | X |
| Hs.31793 | EST | up 5 days | X | X | X |
| Hs.326 | TAR (HIV) RNA binding protein 2 | up 5 days | 0 | 0 | 0 |
| Hs.32989 | receptor (calcitonin) activity modifying protein 1 | up 6 hours | 0 | 0 | 3 |
| Hs.33476 | CasL interacting molecule | down 6 hours | 0 | 0 | 0 |
| Hs.3459 | EST | down 6 hours | X | X | X |
| Hs.36793 | solute carrier family 12 (potassium/chloride transporters), member 8 | down 6 hours | 0 | 0 | 0 |
| Hs.37165 | collagen, type IX, alpha 2 | down 6 hours | 0 | 0 | 0 |
| Hs.38176 | SCN Circadian Oscillatory Protein (SCOP) | down 6 hours | 0 | 0 | 0 |
| Hs.3991 | CDC26 subunit of anaphase promoting complex | up 6 hours & 5 days | 0 | 0 | 0 |
| Hs.4084 | KIAA1025 protein | down 6 hours | 0 | 0 | 0 |
| Hs.42586 | KIAA1560 protein | down 6 hours & 5 days | 0 | 0 | 0 |
| Hs.42959 | KIAA1012 protein | down 6 hours & 5 days | 0 | 0 | 0 |
| Hs.44205 | EST | down 5 days | X | X | X |
| Hs.44344 | hypothetical protein FLJ20534 | down 6 hours | 0 | 0 | 0 |
| Hs.479 | RAB5C, member RAS oncogene family | down 6 hours | 0 | 0 | 0 |
| Hs.49117 | EST | down 5 days | X | X | X |
| Hs.49282 | EST | up 6 hours & 5 days | X | X | X |
| Hs.5085 | dolichyl-phosphate mannosyltransferase polypeptide 1 | up 5 days | 0 | 0 | 0 |
| Hs.5344 | adaptor-related protein complex 1, gamma 1 subunit | down 5 days | 0 | 0 | 0 |
| Hs.54483 | N-myc (and STAT) interactor | up 6 hours | 0 | 0 | 0 |
| Hs.54642 | methionine adenosyltransferase II, beta | down 5 days | 0 | 0 | 0 |
| Hs.55608 | hypothetical protein MGC955 | up 6 hours & 5 days | 0 | 0 | 0 |
| Hs.57304 | Ras-related GTP-binding protein | up 5 days | 1 | 0 | 14 |
| Hs.64322 | SIPL protein | down 6 hours | 0 | 0 | 0 |
| Hs.70186 | suppressor of Ty 5 homolog | up 5 days | 0 | 0 | 0 |
| Hs.74047 | electron-transfer-flavoprotein, beta polypeptide | down 6 hours | 0 | 0 | 0 |
| Hs.74369 | integrin, alpha 7 | down 5 days | 54 | 1 | 272 |
| Hs.74563 | ornithine decarboxylase antizyme 2 | down 5 days | 4 | 1 | 5 |
| Hs.74597 | stromal interaction molecule 1 | up 5 days | 1 | 11 | 0 |
| Hs.75180 | protein phosphatase 5 | down 5 days | 0 | 0 | 0 |
| Hs.75835 | phosphomannomutase 1 | up 6 hours & 5 days | 0 | 1 | 0 |
| Hs.75841 | chromosome 12 open reading frame 8 | down 6 hours | 0 | 0 | 0 |
| Hs.76847 | alpha glucosidase II alpha subunit | up 5 days | 0 | 0 | 0 |
| Hs.76917 | F-box only protein 8 | up 6 hours & 5 days | 0 | 0 | 0 |
| Hs.7739 | hypothetical protein MGC12904 | up 6 hours | 0 | 0 | 0 |
| Hs.78354 | surfeit 5 | down 6 hours & 5 days | 0 | 0 | 0 |
| Hs.78504 | inner membrane protein, mitochondrial (mitofilin) | down 6 hours | 0 | 0 | 0 |
| Hs.79322 | glutaminyl-tRNA synthetase | up 6 hours | 0 | 0 | 1 |
| Hs.79358 | testis-specific kinase 1 | down 6 hours | 0 | 0 | 0 |
| Hs.79440 | IGF-II mRNA-binding protein 3 | up 6 hours | 2 | 1 | 7 |
| Hs.80120 | UDP-N-acetyl-alpha-D-galactosamine:polypeptide N-acetylgalactosaminyltransferase 1 | down 6 hours | 0 | 0 | 0 |
| Hs.808 | heterogeneous nuclear ribonucleoprotein F | up 6 hours & 5 days | 0 | 0 | 32 |
| Hs.8117 | erbb2 interacting protein | up 6 hours & 5 days | 0 | 0 | 0 |
| Hs.82483 | EST | down 6 hours | 0 | 0 | 0 |
| Hs.83023 | peroxisomal biogenesis factor 11B | up 6 hours | 0 | 0 | 0 |
| Hs.83070 | growth factor receptor-bound protein 14 | down 6 hours & 5 days | 0 | 0 | 0 |
| Hs.83954 | protein associated with PRK1 | down 6 hours & 5 days | 0 | 0 | 0 |
| Hs.8518 | EST | up 6 hours | 0 | 0 | 0 |
| Hs.85302 | adenosine deaminase, RNA-specific, B1 | down 5 days | 0 | 0 | 0 |
| Hs.87327 | EST | up 6 hours & 5 days | 0 | 0 | 0 |
| Hs.8834 | ring finger protein 3 | down 5 days | 0 | 0 | 0 |
| Hs.90572 | PTK7 protein tyrosine kinase 7 | up 6 hours | 0 | 0 | 0 |
| Hs.91393 | EST | up 5 days | X | X | X |
| Hs.91481 | NEU1 protein | up 5 days | 0 | 0 | 1 |
| Hs.92002 | guanine nucleotide binding protein (G protein), | up 6 hours | 4 | 8 | 41 |
| Hs.929 | myosin, heavy polypeptide 7, | down 5 days | 0 | 0 | 11 |
| Hs.93678 | hypothetical protein p5326 | up 6 hours | 0 | 0 | 0 |
| Hs.97477 | lysozyme homolog | up 6 hours & 5 days | 0 | 0 | 0 |
